# Retatrutide and lipid and metabolite profiles in participants with obesity with or without type 2 diabetes

**DOI:** 10.1210/clinem/dgag201

**Published:** 2026-05-14

**Authors:** Mackenzie J Pearson, Jill A Willency, Yanzhu Lin, Arkan Abadi, Mark L Hartman, Tamer Coskun, Giacomo Ruotolo, Kevin L Duffin, Axel Haupt, Christopher B Newgard, Valentina Pirro

**Affiliations:** Cardiometabolic Research, Eli Lilly and Company, Indianapolis, IN 46225, USA; Cardiometabolic Research, Eli Lilly and Company, Indianapolis, IN 46225, USA; Cardiometabolic Research, Eli Lilly and Company, Indianapolis, IN 46225, USA; Cardiometabolic Research, Eli Lilly and Company, Indianapolis, IN 46225, USA; Cardiometabolic Research, Eli Lilly and Company, Indianapolis, IN 46225, USA; Cardiometabolic Research, Eli Lilly and Company, Indianapolis, IN 46225, USA; Cardiometabolic Research, Eli Lilly and Company, Indianapolis, IN 46225, USA; Cardiometabolic Research, Eli Lilly and Company, Indianapolis, IN 46225, USA; Cardiometabolic Research, Eli Lilly and Company, Indianapolis, IN 46225, USA; Duke Molecular Physiology Institute and Sarah W. Stedman Nutrition and Metabolism Center, Duke University School of Medicine, Durham, NC 27705, USA; Department of Medicine, Division of Endocrinology, Metabolism, and Nutrition, Duke University School of Medicine, Durham, NC 27705, USA; Department of Pharmacology and Cancer Biology, Duke University Medical Center, Durham, NC 27705, USA; Cardiometabolic Research, Eli Lilly and Company, Indianapolis, IN 46225, USA

**Keywords:** multihormone receptor agonist, glucagon, ketone, carnitine, lipid metabolism

## Abstract

**Context:**

In phase 2 trials, retatrutide reduced body weight, hemoglobin A1c and improved the lipid profiles of participants living with obesity, with and without T2D.

**Objective:**

Assess plasma metabolome and lipidome changes associated with retatrutide treatment.

**Methods:**

Metabolomics and lipidomics were performed in fasting samples from 2 randomized, placebo-controlled phase 2 trials as a post hoc exploratory analysis. Participants living with obesity with and without T2D were treated for 36 and 48 weeks, respectively. There were 282 and 213 participants in the obesity and T2D trials, respectively and the intervention(s) received were: Obesity trial: retatrutide (1, 4, 8, and 12 mg) or placebo; T2D trial: retatrutide (0.5, 4, 8, and 12 mg) or placebo or dulaglutide (1.5 mg). The main outcome measure(s) were changes in metabolite and lipid levels with retatrutide treatment against baseline levels and placebo using multiplicity correction.

**Results:**

At both primary and study endpoints for both populations, higher doses of retatrutide were associated with changes in a cluster of metabolites comprising 3-hydroxybutyrate, acetylcarnitine, free carnitine, and fatty acid–derived long-chain acylcarnitines. Mediation analyses suggested that changes in these biomarkers mediated 23.2% of the weight reduction response in participants without T2D and that this mediation was blunted to 12.7% in participants with T2D. Retatrutide treatment was also associated with changes in metabolites associated with insulin resistance, including branched-chain amino acids and their catabolic products, 2-aminoadipic acid, 2-hydroxybutyrate, urate, and triglycerides enriched in short-chain and saturated acyl side chains. These changes were found in both study populations and sustained across study endpoints.

**Conclusion:**

Retatrutide was associated with changes in 2 metabolic clusters related to fatty acid oxidation and insulin resistance in a direction associated with improved metabolic health and reduced cardiovascular risk.

Retatrutide is a synthetic molecule with activity at the glucose-dependent insulinotropic polypeptide (GIP), glucagon-like peptide 1 (GLP-1), and glucagon (GCG) receptors. Retatrutide is under investigation for the treatment of obesity and type 2 diabetes (T2D) (1). By engaging several receptors with a single agent, retatrutide may modulate energy balance, weight reduction, and glycemic control simultaneously, with beneficial effects on insulin signaling, amino acid metabolism, and lipid homeostasis (2-7).

In a phase 2 trial involving adults with obesity, treatment with retatrutide resulted in a mean weight reduction of up to 24.2% after 48 weeks for the highest dose tested. Additionally, participants showed a significant reduction in waist circumference (8). MRI scans performed in a substudy of participants with metabolic dysfunction–associated steatotic liver disease (MASLD) demonstrated that the reported weight reduction and decrease in waist circumference were associated with almost complete clearance of fat from the liver, reduction in liver volume, and a decrease in the visceral adipose depot volume (9). At weeks 24 and 48, treatment with retatrutide was associated with improvements in exploratory cardiometabolic endpoints, including systolic and diastolic blood pressure, glycated hemoglobin (HbA1c), fasting glucose and insulin levels, homeostatic model assessment for insulin resistance (IR), fasting lipid parameters including triglycerides (TGs), total cholesterol, low-density lipoprotein (LDL) cholesterol, and very low-density lipoprotein (VLDL) cholesterol (8).

In a second phase 2 study that enrolled adults living with T2D, retatrutide treatment resulted in significant weight reduction over a 36-week period, with an average body weight reduction of up to 16.9%. Retatrutide treatment also showed clinically meaningful improvements in glycemic control, with an average reduction in Hb1Ac (%) of up to 2.16% (HbA1c [mmol/mol] change from baseline of −23.59) for the 12-mg group after 36 weeks. Treatment of the T2D cohort with retatrutide was also associated with improvements in TGs, non-HDL cholesterol, and markers of insulin sensitivity (10). In both phase 2 trials, treatment with retatrutide exhibited a safety profile generally comparable to that of other incretin-based therapies (8, 10).

Although the mechanisms of action have not been fully elucidated, the increased weight reduction and glycemic control observed with retatrutide treatment vs other incretin-based therapies are hypothesized to derive from the combined effects of GCG receptor agonism to increase energy expenditure (11, 12), GLP-1R agonism to decrease energy intake by controlling central satiety (13) and GIP receptor agonism to improve adipose tissue health, peripheral insulin sensitivity, and whole-body energy metabolism (14-16). In obese mice, treatment with retatrutide was shown to reduce food intake, increase energy expenditure, and increase fatty acid oxidation (FAO), as illustrated by a reduction in respiratory exchange ratio, resulting in significant weight reduction. The use of a GCG receptor antibody antagonist in combination with retatrutide treatment did not affect food intake, but blocked the effect on energy expenditure, confirming a role for GCG receptor agonism in this response (1).

Since reports of in-trial outcomes showed an amelioration of obesity-related disease phenotypes after treatment with retatrutide (8, 10), in this post hoc analysis study, we aimed to understand the biochemical changes underlying the metabolic health improvements observed with retatrutide treatment in participants living with obesity with or without T2D. Toward that end, we measured metabolite and lipid changes by targeted mass spectrometry.

We report 2 prominent metabolic signatures that changed with retatrutide treatment: one associated with activation of FAO and the second associated with improved IR. Taken together, our findings show retatrutide-associated changes in lipid homeostasis and metabolic profile that are consistent with amelioration of key features of IR associated with obesity and T2D pathogenesis.

## Materials and methods

### Participants and study design

A full description of the study methods, and efficacy and safety results has been published (8, 10). Briefly, a phase 2, double-blind, randomized, placebo-controlled trial enrolled adults who had a BMI of 30 kg/m^2^ or higher or who had a BMI of 27 kg/m^2^ to <30 kg/m^2^ with at least one weight-related condition (referred to as the obesity trial). Participants were randomly assigned to receive subcutaneous maintenance doses of retatrutide (1, 4, 8, or 12 mg) or placebo once weekly for 48 weeks (Fig. S1 (17)) (8).

A phase 2, double-blind, double-dummy, placebo-controlled, and active comparator-controlled trial enrolled adults with T2D, HbA1c of 7.0-10.5% (53.0-91.3 mmol/mol) and BMI of 25-50 kg/m^2^, treated with diet and exercise alone or with a stable dose of metformin for at least 3 months prior to trial enrollment (referred to as the T2D trial). Participants were randomly assigned to receive once weekly subcutaneous injections of placebo, dulaglutide 1.5 mg, or retatrutide maintenance doses of 0.5, 4, 8, or 12 mg once weekly for 36 weeks (Fig. S2 [17]) (10).

### Study procedures

For the obesity trial, fasting plasma samples were collected at baseline, 24, and 48 weeks posttreatment. For the T2D trial, fasting plasma samples were collected at baseline, 24, and 36 weeks posttreatment. Sample collection followed standardized trial protocol guidelines, and all samples are assumed to have been processed under homogenous and controlled conditions up to freezing at −80 °C. All available samples used for post hoc exploratory omics analyses had been thawed once for aliquoting and then refrozen and stored at −80 °C until ready for liquid chromatography-tandem mass spectrometry (LC-MS/MS) analysis.

### Study approvals

The phase 2 trials were registered at ClinicalTrials.gov with numbers NCT04881760 (obesity trial) and NCT04867785 (T2D trial). Trials were conducted in accordance with the Declaration of Helsinki and the Council for International Organizations of Medical Sciences International Ethical Guidelines, the International Conference on Harmonization Good Clinical Practices Guideline, and all other applicable laws and regulations. All participants provided written consent prior to participation in the study.

### Targeted LC-MS/MS analyses

Independent aliquots of plasma samples were processed and surveyed using 3 mass spectrometry–based approaches targeting small polar metabolites (below 500 Da), complex lipid species, and lipid mediators, respectively. The methodologies have been previously described (18-20). A summary can be found in the supplemental material (17).

For most of the participants (282 and 213 for the obesity and T2D trial, respectively), sample availability was sufficient to perform all omics analyses. We note that for a small number of participants, sample availability was limited and allowed only 1 or 2 of the omics analyses to be performed. The exact number of participants used for any statistical test is detailed in the “Results” section.

### Statistical analysis

Participants from the modified intention-to-treat population, ie, without data after study drug discontinuation or rescue drug initiation, were included in this analysis. Only patients with nonmissing baseline values were included. Data were assessed using mixed models for repeated measures that included treatment, time, and treatment-by-time interaction. Log transformation was applied before the model fitting to improve data normality. Data from participants following different titration schemes for the 4 mg and the 8 mg maintenance dose arms were pooled. Statistical test results were considered significant at false discovery rate adjusted *P*-value (FDR Adj. *P*) <.05, unless otherwise noted. Spearman's rank correlation was used to explore the association between the biomarkers themselves and against weight. Correlations were computed on percent change from baseline and considered significant for *P*-values <.05, unless otherwise noted. Weight reduction data used for correlation analysis were collected as part of the original phase 2 studies (8, 10). Statistical analyses were implemented by using R 3.6.0, Prism 10 (San Diego, CA, USA) and SAS 9.4 software (Cary, NC, USA).

Hierarchical clustering analysis on metabolomics datasets was conducted with the “clustergram” function in MATLAB (version 2019b, MathWorks) (21). MetaboAnalyst 6.0 was employed for metabolite set enrichment analysis.

Mediation analysis was conducted to estimate the mediation effect sizes (individual and joint) of change from baseline in specific metabolites and metabolite ratios at 24 weeks posttreatment on percent change from baseline body weight at study endpoint (48 and 36 weeks posttreatment for the obesity and T2D trials, respectively). For the obesity trial, the mediation effect sizes of biomarkers (24 weeks posttreatment) were also estimated for weight reduction at 36 weeks, so that results could be compared over the same duration of treatment across both studies. For the mediation analyses, retatrutide 8 and 12 mg arms were pooled and compared to placebo. A directed acyclic graph illustrating the hypothesized mediation structure is presented with the results. The analysis estimated the total effect size of treatment exposure on the outcome (change from baseline body weight at endpoint), the individual and joint indirect effect sizes mediated through the biomarkers at 24 weeks (log ratio change from baseline), and the direct effect sizes of treatment not mediated by these biomarkers. Inference was performed using nonparametric bootstrapping. The mean of the bootstrapped estimates (5000), along with bias-corrected and accelerated confidence intervals and quantile-based *P*-values, are reported. Mediation analyses were conducted with the “mma” package in R (22, 23). If figures were edited using Adobe Photoshop CC 2024, it is indicated in the legends.

## Results

### Metabolic changes following retatrutide treatment in the obesity trial population

Targeted LC-MS/MS assays measured 143 small polar metabolites in fasting plasma from 282 participants in the obesity trial at baseline, 24, and 48 weeks (820 samples). Global lipidomic analysis surveyed 566 individual lipid species across 12 classes, including acyl chain length and saturation, in 813 samples from the same participants. The sample population analyzed by lipidomics overlapped entirely with the one that underwent targeted metabolomics (Table S1 [17]). Baseline patient characteristics were similar across all treatment groups (Table S1 [17]).

Two metabolic clusters emerged after 24 weeks of retatrutide treatment (Fig. 1): one associated with FAO activation and the other with amelioration of IR. We highlight below the 24-week changes of specific metabolites associated with both clusters and their remodeling observed after 48 weeks. A high-level summary of the total number of metabolites and lipids that showed significant changes associated with treatment, both against baseline levels and placebo, is presented in the supplemental results section (17).

**Figure 1:**
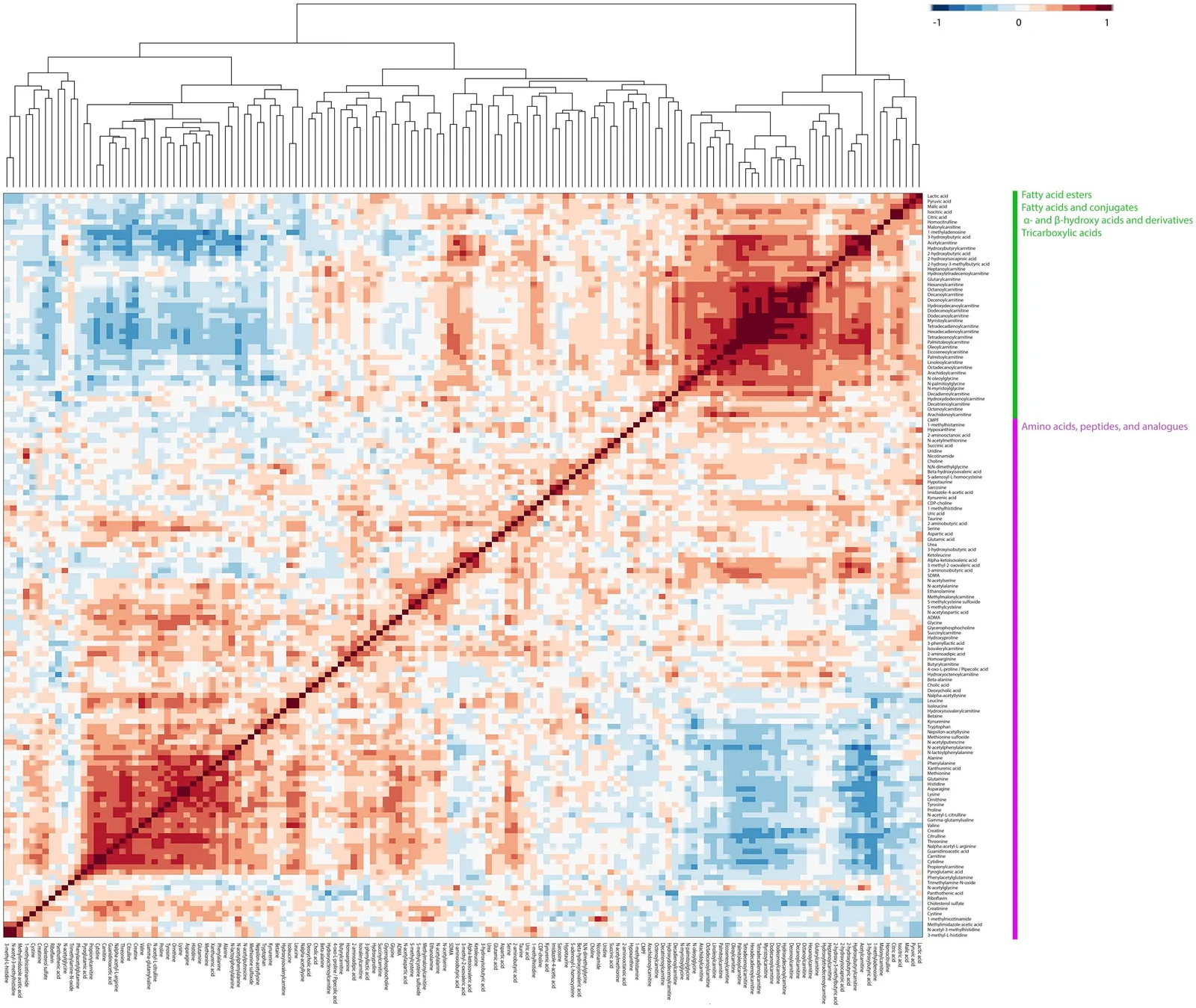
Correlation heatmap of percent change from baseline across all metabolites measured for retatrutide 12 mg at 24-week primary endpoint in the obesity trial population. Hierarchical clustering analysis was applied, and the returned dendrogram is displayed. Clustergram function was performed using MATLAB version 2019b (MathWorks). MetaboAnalyst 6.0 was employed for metabolite set enrichment analysis. The chemical subclasses of the clustered metabolites are highlighted as the right inset, which was edited into the figure using Adobe Photoshop 2024.

### Twenty-four-week changes of intermediates of FAO in the obesity trial population

The metabolite with the largest magnitude of change from baseline was the ketone body 3-hydroxybutyric acid (3-HB). The levels increased significantly compared to both baseline and placebo for retatrutide doses 4 mg and higher (Fig. 2A), with a maximal average percent change from baseline of 198% with retatrutide 12 mg. This result in the larger population trial aligns with previous findings in the MASLD subpopulation, in which targeted measurement of 3-HB revealed a 2- to 3-fold increase in its plasma levels relative to baseline in a dose-related pattern, with retatrutide doses 4 mg and higher (9).

**Figure 2:**
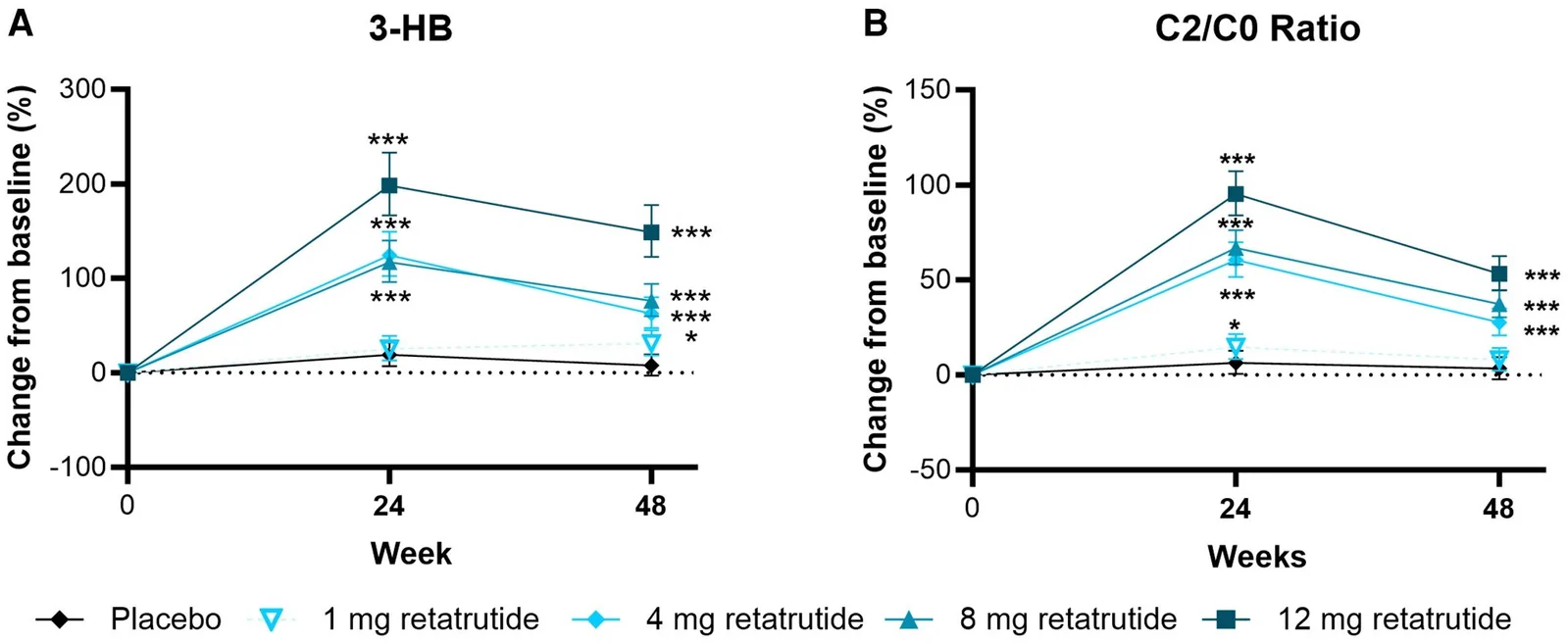
Data are from modified intention-to-treat population (obesity trial). For all treatment group and timepoints, least square mean of percent change from baseline ± standard error for (A) 3-HB (24-week percent change from baseline [FDR-adjusted *P*-value vs placebo, *P*]: Placebo; 19.1; retatrutide 1, 4, 8, and 12 mg: 25.6 [*P* > .05], 124.6 [*P* < .001], 117.0 [*P* < .001], and 198.0 [*P* < .001]; 48-week percent change from baseline: Placebo, 7.8; retatrutide 1, 4, 8, and 12 mg, 31.1 [*P* > .05], 62.72 [*P* < .05], 76.1 [*P* < .01], and 148.7 [*P* < .001]) and (B) C2/C0 ratio (24-week percent change from baseline: Placebo, 6.4; retatrutide 1, 4, 8, and 12 mg, 14.8 [*P* < .05], 60.5 [*P* < .001], 66.9 [*P* < .001], and 95.3 [*P* < .001]; 48-week percent change from baseline: Placebo 3.4; retatrutide 1, 4, 8, and 12 mg, 8.0 [*P* > .05], 27.7 [*P* < .001], 37.2 [*P* < .001], and 53.3 [*P* < .001]). *P < .05*, **P < .01, ***P < .001 vs baseline. *P* ≥ .05 is considered not significant. Abbreviations: 3-HB, 3-hydroxybutyric acid; C0, free carnitine; C2, acetylcarnitine; FDR, false discovery rate.

The 3-HB percent change from baseline was significantly and inversely correlated with 24-week weight reduction across all retatrutide doses (Spearman's rho = −0.3, −0.6, −0.4, and −0.4 for retatrutide 1, 4, 8, and 12 mg respectively, *P*-value <.05). Figure S3 (17) shows the mean log ratio change of 3-HB per quartile of weight reduction for the combined retatrutide 8 and 12 mg groups, since the weight reduction was not statistically different between the 2 doses at the primary endpoint. The 48-week weight reduction also correlated with 24-week 3-HB increases (Spearman's rho = −0.4, −0.7, −0.4, and −0.5 for retatrutide 1, 4, 8, 12 mg respectively), as well as with the levels of 3-HB at 24 weeks (Spearman's rho = −0.4, −0.7, −0.4, and −0.5 for retatrutide 1, 4, 8, and 12 mg respectively, *P*-value <.05), but did not correlate significantly with the baseline ketone levels (*P*-values ≥.05).

The 3-HB increase was accompanied by significant elevation of 3-hydroxybutyrylcarnitine (C4OH) levels for retatrutide 4, 8, and 12 mg (Table S2 [17]). Metabolite C4OH is formed from L-3-hydroxybutyryl coenzyme A (CoA), a FAO intermediate, and can also be produced from 3-HB (24).

Consistent with increased FAO, retatrutide was associated with a rise in acetylcarnitine (C2), a surrogate of the acetyl-CoA pool, at doses 4, 8, and 12 mg (Table S2 [17]). We note that the increase in the C2 pool was associated with increases in citrate and isocitrate in response to retatrutide 8 and 12 mg (citrate mean percent change from baseline of 21.4% and 10.9% for retatrutide 12 and 8 mg, respectively [FDR Adj. *P* < .01]; isocitrate mean percent change from baseline of 11.2% and 13.7% for retatrutide 12 and 8 mg, respectively [FDR Adj. *P* = .052 and .0071]). Both are early intermediates in the tricarboxylic acid or Krebs cycle generated in part from acetyl-CoA.

Inversely, we observed a significant dose-dependent decrease of free carnitine (C0) compared to baseline and placebo (Table S2 [17]). In states of increased FAO, C0 is consumed for synthesis of long-chain acylcarnitines (ACs) via the carnitine shuttle (25). Indeed, with retatrutide treatment, we observed significant increases in circulating levels of various ACs ranging in length from C8 to C18 (Table S2 [17]).

We used the ratio of C2 carnitine to C0 (C2/C0) as an indicator of fatty acid utilization as fuel source (26). Figure 2B shows increases in the C2/C0 ratio in a retatrutide dose-dependent fashion compared to baseline levels, with a peak increase of 95.3% for retatrutide 12 mg. As reported for 3-HB, the 24-week percent change from baseline for C2/C0 ratio was significantly and inversely correlated with the 24-week weight reduction for retatrutide 4 mg and higher (Spearman's rho = −0.5, −0.4, and −0.6 for retatrutide 12, 8, and 4 mg respectively, *P*-values <.05). Figure S3 (17) shows the stratification of the 24-week log ratio change of C2/C0 per quartile of the 24-week weight reduction for the combined 2 higher doses of retatrutide.

### Twenty-four-week changes of metabolite clusters associated with IR in the obesity trial population

The second cluster of metabolites emerging from the omics platforms deployed in this study have been robustly associated with IR and related cardiometabolic disease phenotypes, including changes in circulating TGs, branched-chain amino acids (BCAAs), 2-aminoadipic acid (2-AAA), and uric acid.

Lipidomic profiling characterized 93 individual TG species by acyl chain length and saturation (Fig. 3). Although TG species across all carbon lengths (46-58 total carbons) decreased significantly with retatrutide 4 mg and higher (Table S3 [17]), the stratification revealed a larger decrease of species containing shorter fatty acids. The TGs were also stratified by their content of biologically relevant fatty acid residues such as myristic (14:0), palmitic (16:0), palmitoleic (16:1), stearic (18:0), oleic (18:1), linoleic (18:2), arachidonic (20:4), and docosahexaenoic (22:6) acids. While all species decreased with retatrutide 4 mg and higher, TGs containing saturated fatty acids showed a greater reduction from baseline compared to those containing unsaturated fatty acids (Table S3 [17]).

**Figure 3:**
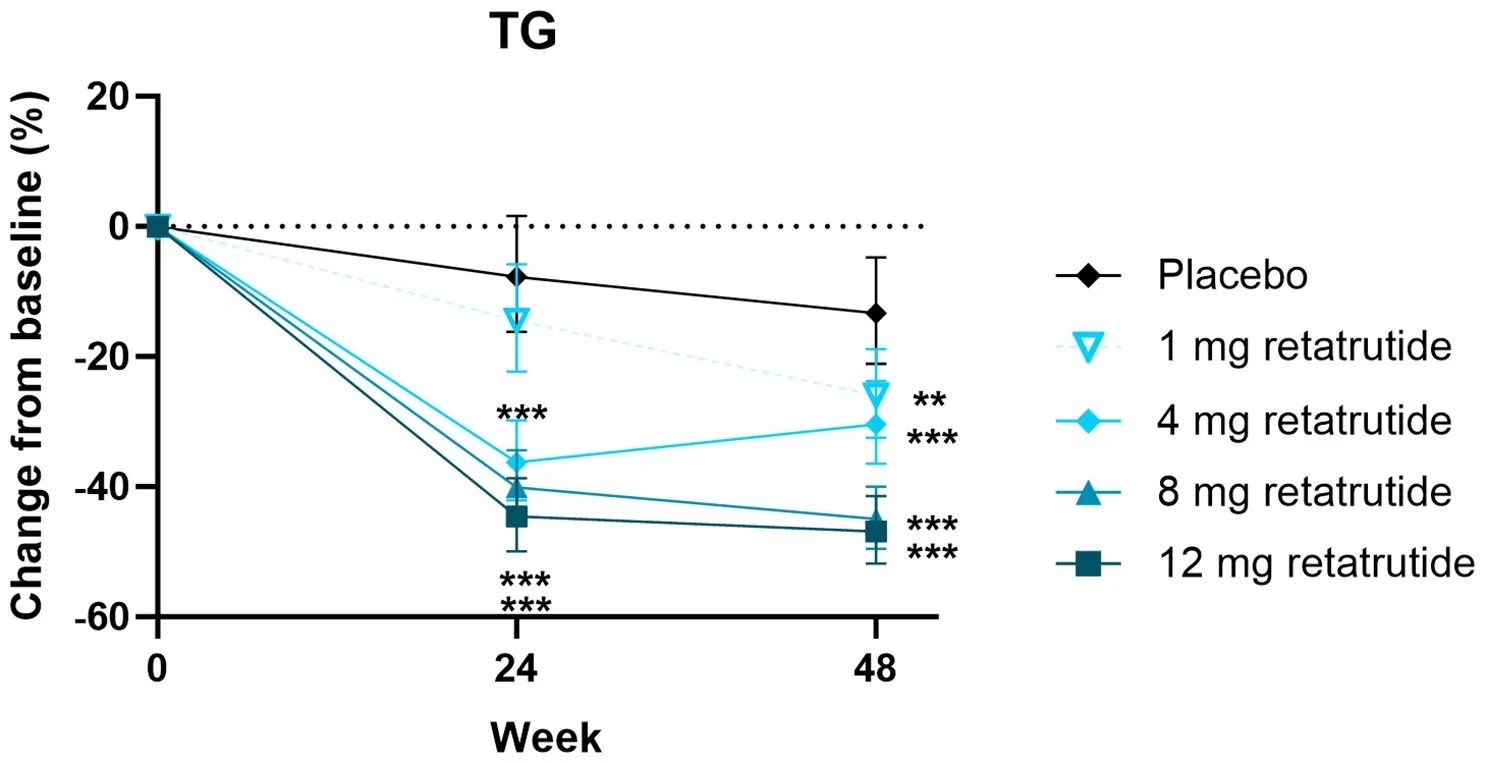
Data are from the modified intention-to-treat population (obesity trial). Least square mean of percentage change from baseline ± standard error of TG with all treatment groups at 24 and 48 weeks. FDR-adjusted *P*-value: *P < .05, **P < .01, ***P < .001 vs baseline. FDR-adjusted *P* ≥ .05 is considered not significant. For total TGs, 24-week percent change from baseline: Placebo, −7.7; retatrutide 1, 4, 8, and 12 mg, −14.4 (*P* > .05), −36.24 (*P* < .01), −40.1 (*P* < .01), and −44.6 (*P* < .001). Forty-eight-week percent change from baseline: Placebo, −13.3; retatrutide 1, 4, 8, and 12 mg, −26.0 (*P* > .05), −30.4 (*P* > .05), −45.0 (*P* < .001), and −46.9 (*P* < .001). Abbreviations: FDR, false discovery rate; TGs, triglycerides.

Retatrutide was also associated with a dose-dependent decrease in circulating BCAAs and related metabolites (Table 1), with the largest decrease in valine at 12 mg. BCAA catabolic products (glutamic acid, ketoleucine, ketovaline, and 3-hydroxyisobutyric acid [3-HIB]) also decreased, as did short-chain ACs malonylcarnitine (C3DC), propionylcarnitine (C3), and isovalerylcarnitine (C5); the latter 2 derived from BCAA catabolism. This contrasts with the increases in FAO-related ACs (Table S2 [17]).

**Table 1:** Twenty-four- and 48-week percent change from baseline of BCAAs, glutamate, BCKAs, 3-HIB, 2-HB, 2-AAA, and urate observed in the obesity trial

	Retatrutide 12 mg	Retatrutide 8 mg	Retatrutide 4 mg	Retatrutide 1 mg	Placebo
Week 24					
BCAAs					
Isoleucine	−21.1*^c,d^* (−18.2 ÷ −24)	−18.2*^c,d^* (−15.4 ÷ −20.9)	−6.4 (−3.1 ÷ −9.7)	0.4 (3.9 ÷ −3.0)	4.5 (8.3 ÷ 0.9)
Leucine	−27.9*^c,d^* (−25.3 ÷ −30.3)	−24.2*^c,d^* (−21.8 ÷ −26.6)	−11.1*^c,d^* (−8.1 ÷ −14.0)	−4.2*^d^* (−1 ÷ −7.2)	2.5 (5.9 ÷ −0.8)
Valine	−32.5*^c,d^* (−30.3 ÷ −34.7)	−26.8*^c,d^* (−24.6 ÷ −29.0)	−14.8*^c,d^* (−12.1 ÷ −17.5)	−3.7 (−0.6 ÷ −6.6)	2.3 (5.6 ÷ −0.8)
BCAA catabolic products					
3-HIB	−11.0*^a,d^* (−6.8 ÷ −15.0)	−8.9*^a,d^* (−5.2 ÷ −12.5)	−9.0*^a,d^* (−5.2 ÷ −12.6)	−6.5 (−2.6 ÷ −10.3)	12.0 (16.8 ÷ 7.4)
Glutamate	−49.2*^c,d^* (−45.9 ÷ −52.4)	−44.1*^c,d^* (−40.7 ÷ −47.2)	−36.0*^c,d^* (−32 ÷ −39.8)	−14.8*^b^* (−9.5 ÷ −19.8)	−3.4 (2.7 ÷ −9.1)
KIC	−11.1*^b^* (−7.6 ÷ −14.6)	−7.4*^a^* (−4.1 ÷ −10.5)	−2.9 (0.6 ÷ −6.2)	−1.2 (2.4 ÷ −4.5)	−0.7 (2.9 ÷ −4.2)
KIV	−13.9*^c,d^* (−11.2 ÷ −16.5)	−9.3*^c^* (−6.8 ÷ −11.7)	−7.3*^a^* (−4.8 ÷ −9.8)	−5.5 (−2.9 ÷ −8.0)	−4.7 (−2.0 ÷ −7.4)
KMV	−2.1 (1.7 ÷ −5.7)	−2.8 (0.5 ÷ −6.0)	−1.2 (2.2 ÷ −4.5)	−1.5 (1.9 ÷ −4.8)	−2.6 0.8 ÷ −6.0
Other metabolites					
2-AAA	−41.4*^c,d^* (−38.5 ÷ −44.1)	−35.1*^c,d^* (−32.2 ÷ −37.8)	−26.5*^c,d^* (−23.1 ÷ −29.8)	−9.3*^a^* (−5.1 ÷ −13.3)	7.7 (12.7 ÷ 2.9)
2-HB	0.0 (6.6 ÷ −6.2)	−9.5 (−4.2 ÷ −14.4)	−1.5 (4.3 ÷ −7.0)	−1.3 (4.5 ÷ −6.8)	7.4 (13.9 ÷ 1.2)
Urate	−22.1*^c,d^* (−18.8 ÷ −25.2)	−16.3*^c,d^* (−13.2 ÷ −19.3)	−13.9*^c,d^* (−10.7 ÷ −17.0)	−6.8 (−3.3 ÷ −10.1)	−0.7 (3.1 ÷ −4.4)
Week 48					
BCAAs					
Isoleucine	−20.0*^c,d^* (−16.9 ÷ −23.1)	−16.0*^c,d^* (−13.1 ÷ −18.8)	−15.4*^c,d^* (−12.3 ÷ −18.4)	−2.6 (1.0 ÷ −6.1)	4.4 (8.3 ÷ 0.6)
Leucine	−22.6*^c,d^* (−19.9 ÷ −25.3)	−20.2*^c,d^* (−17.8 ÷ −22.6)	−16.2*^c,d^* (−13.5 ÷ −18.9)	−4.0 (−0.8 ÷ −7)	2.2 (5.6 ÷ −1.2)
Valine	−26.5*^c,d^* (−23.9 ÷ −28.9)	−22.3*^c,d^* (−20.0 ÷ −24.6)	−16.9*^c,d^* (−14.3 ÷ −19.5)	−4.5 (−1.5 ÷ −7.5)	−0.7 (2.6 ÷ −3.9)
BCAA catabolic products					
3-HIB	−17.4*^b^* (−12.8 ÷ −21.8)	−15.9*^c^* (−11.9 ÷ −19.7)	−12.0*^a^* (−7.8 ÷ −16.1)	−8.1 (−3.7 ÷ −12.3)	−3.9 (1.0 ÷ −8.6)
Glutamate	−48.0*^c,d^* (−43.8 ÷ −52.0)	−48.7*^c,d^* (−45.0 ÷ −52.1)	−36.6*^c,d^* (−31.8 ÷ −41.1)	−6.0 (1.1 ÷ −12.6)	−4.4 (3.0 ÷ −11.4)
KIC	−16.7*^c^* (−12.9 ÷ −20.4)	−15.6*^c^* (−12.3 ÷ −18.9)	−11.7*^b^* (−8.1 ÷ −15.1)	−7.5 (−3.8 ÷ −11.1)	−11.7 (−7.9 ÷ −15.3)
KIV	−19.2*^c^* (−16.4 ÷ −22.0)	−16.9*^c^* (−14.4 ÷ −19.3)	−12.5*^c^* (−9.8 ÷ −15.0)	−8.2*^a^* (−5.4 ÷ −10.8)	−10.7*^a^* (−7.8 ÷ −13.5)
KMV	−13.4*^b^* (−9.7 ÷ −17.0)	−10.7*^b^* (−7.4 ÷ −13.9)	−10.3*^b^* (−7.0 ÷ −13.6)	−7.2 (−3.7 ÷ −10.5)	−8.8 (−5.2 ÷ −12.3)
Other metabolites					
2-AAA	−36.4*^c,d^* (−32.9 ÷ −39.6)	−31.5*^c,d^* (−28.3 ÷ −34.7)	−24.2*^c,d^* (−20.4 ÷ −27.9)	−8.6 (−4.0 ÷ −13.0)	6.4 (11.9 ÷ 1.1)
2-HB	−16.3*^a^* (−10.2 ÷ −21.9)	−23.1*^c^* (−18.3 ÷ −27.6)	−16.2*^a^* (−10.8 ÷ −21.2)	−11.4 (−5.8 ÷ −16.6)	−8.7 (−2.6 ÷ −14.5)
Urate	−26.5*^c,d^* (−23.8 ÷ −29.1)	−15.9*^c,d^* (−13.2 ÷ −18.5)	−16.6*^c,d^* (−13.9 ÷ −19.2)	−9.6*^a^* (−6.7 ÷ −12.4)	−5.8 (−2.6 ÷ −9.0)

Data are presented as LSM of percentage change (1 SE upper one ÷ 1 SE lower) from the modified intention-to-treat population. FDR-adjusted *P*-values: *^a^P*<.05; *^b^P*<.01; *^c^*P<.001 compared to baseline; *^d^P*<.05 compared to placebo.

Abbreviations: 2-AAA, 2-aminoadipic acid; 2-HB, 2-hydroxybutyric acid; 3-HIB, 3-hydroxyisobutyric acid; BCAA, branched-chain amino acid; BCKA, branched-chain ketoacids; FDR, false discovery rate; KIC, α-ketoisocaproate; KMV, α-keto-β-methylvalerate; KIV, α-ketoisovalerate; LSM, least square mean.

Retatrutide was also associated with significant lowering of several other circulating amino acids (Table S4 [17]), consistent with GCG receptor-mediated stimulation of gluconeogenesis and ureagenesis (27). For example, alanine levels were lowered up to mean percent change from baseline of −53.3% with retatrutide 12 mg. In addition, ornithine, citrulline, and aspartic acid, key amino acids involved in the urea cycle, were reduced in circulation with treatment (Table 1). We also noted robust lowering of lysine and methionine levels, which are precursors for C0 and 2-AAA biosynthesis. Concordantly, circulating 2-AAA levels decreased with retatrutide 4 mg and higher compared to baseline and placebo (Table 1). Lastly, we observed significant reductions in circulating uric acid levels compared to both baseline and placebo for retatrutide 4 mg and higher (Table 1).

### Metabolic remodeling following 48 weeks of retatrutide treatment in the obesity trial population

Metabolites modulated at 24 weeks remained altered at 48 weeks, but with attenuated FAO-related responses. In contrast, IR-associated metabolite and lipid changes were conserved or enhanced at 48 weeks.

3-HB remained the metabolite with the largest percent increase from baseline, though attenuated by roughly a third to half compared to 24 weeks (Fig. 2A). C2 and C4OH changes paralleled the 3-HB pattern with reduced magnitude (Table S2 [17]). Citrate and isocitrate changes were no longer significant. Mid- and long-chain ACs, octanoyl- (C8), decanoyl- (C10), dodecanoyl- (C12), tetradecadienoyl- (C14:2), tetradecenoyl- (C14:1), and hexadecadienoyl- (C16:2) carnitines showed significant increases only for retatrutide 12 mg with blunted responses at study endpoint; other ACs were not different from or lower than baseline (Table S2 [17]). The reduction in C0 persisted at 48 weeks and was significantly different from baseline at all retatrutide doses (Table S2 [17]). Consequently, the C2/C0 ratio increase remained significant at 48 weeks compared to baseline (Fig. 2B). We note, however, that neither the 3-HB 48-week percent change from baseline nor the C2/C0 ratio 48-week percent change from baseline correlated with the 48-week weight reduction consistently (*P*-values ≥.05, except for 3-HB with retatrutide 4 mg (Spearman's rho = −0.40 [*P*-value = .0056]) and for C2/C0 with retatrutide 8 mg (Spearman's rho = −0.28 [*P*-value = .032]), unlike previously highlighted for the 24-week percent change from baseline.

Mediation analysis differentiated direct treatment effects from indirect effects mediated by 24-week changes in 3-HB and C2/C0 on 48-week body weight. Individually, the mediation effect size of C2/C0 ratio was observed to be significant, while 3-HB did not show significance. We theorize that this result aligns with C0 and C2 being directly tied to the transport and use of fatty acids as mitochondrial energy substrate, whereas ketogenesis is a byproduct of FAO which provides an alternative pathway for acetyl-CoA disposal and homeostasis. Nevertheless, we note the joint mediation effect size for 3-HB and C2/C0 ratio was significant, directionally synergistic, and estimated to mediate 23.2% of the weight reduction with retatrutide (Fig. 4A and 4C recapitulates the mediation structure).

**Figure 4:**
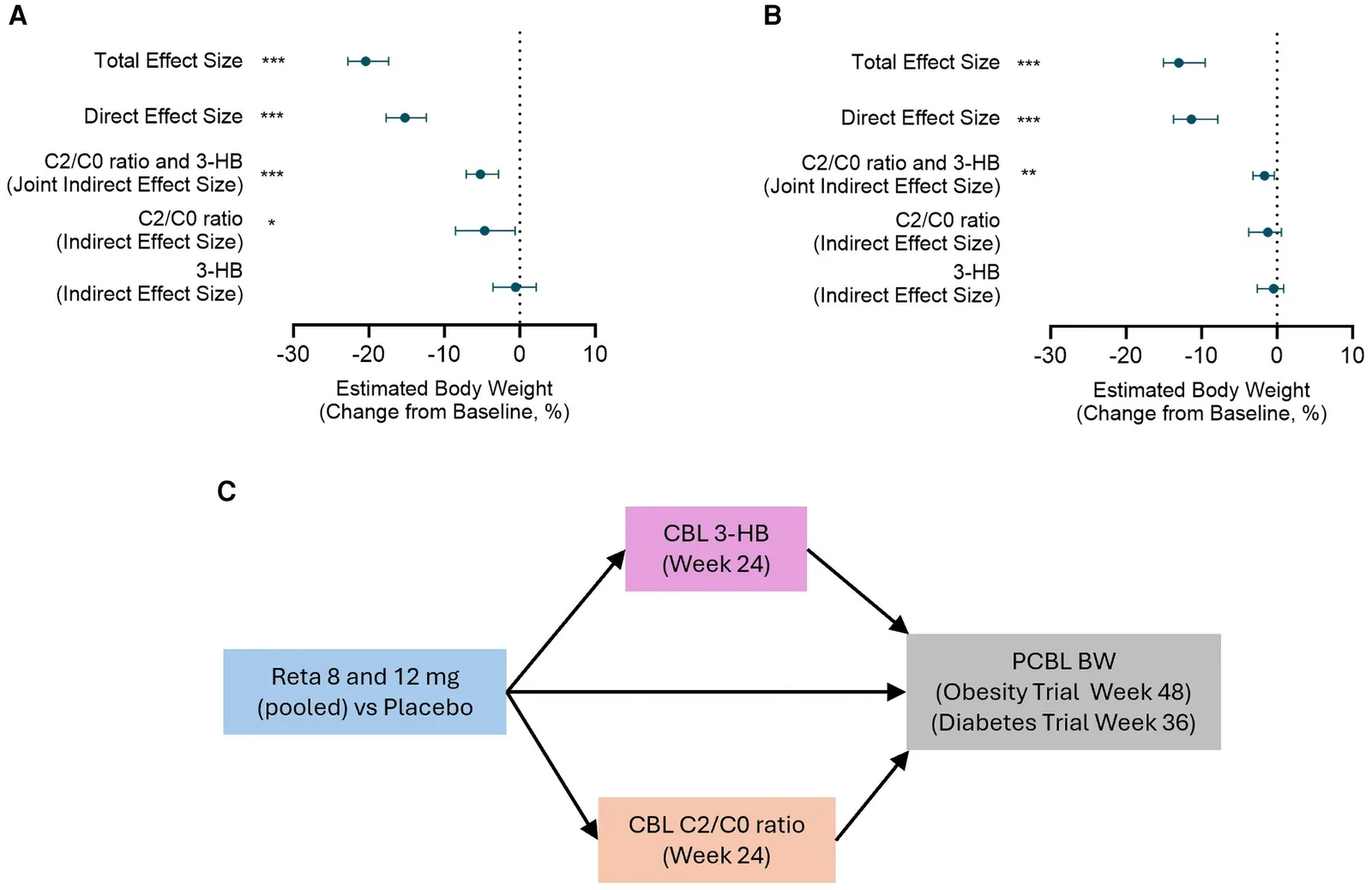
(A) Results from the mediation analysis of the obesity trial with 48-week study endpoint. (B) Results from the mediation analysis of the T2D trial with 36-week study endpoint. Estimates from multiple mediation analysis including total effect size of treatment, the indirect mediation effect size of 3-HB at 24 weeks, the indirect mediation effect size of C2/C0 ratio at 24 weeks, the joint indirect mediation effect size of both 3-HB and C2/C0 at 24 weeks, and the direct effect size of treatment not mediated by these biomarkers are plotted. Plotted are the mean estimates from nonparametric bootstrap, bias-corrected, and accelerated 95% confidence intervals and quantile *P*-values. *P < .05, **P < .01; ***P < .001. (C) The directed acyclic graph of the hypothesized multiple mediation structure with baseline exposure to retatrutide treatment (8 and 12 mg pooled) or placebo treatment, mediators log-CBL 3-HB and C2/C0 at 24 weeks posttreatment, and body weight percent change from baseline (PCBL) reduction at endpoint. Abbreviations: 3-HB, 3-hydroxybutyric acid; BW, body weight; C0, free carnitine; C2, acetylcarnitine; CBL, change from baseline; PCBL, percent change from baseline.

In contrast to the milder effects of retatrutide on the FAO cluster at 48 weeks, TG lowering was conserved at 48 weeks (Fig. 3), with continued preferential decreases in TGs with shorter and more saturated fatty acids. Maximal changes for retatrutide 12 mg ranged between −71.9% for TG 46C and −36.8% for TG 58C, and −66.9% for TG species containing myristic acid (14:0) and −42.4% for species containing arachidonic acid (20:4); see Table S3 (17).

Consistent reductions in plasma amino acid levels were also observed at week 48 compared to baseline. Table 1 details the changes for isoleucine, leucine, valine, and their catabolic products. Changes in 2-AAA at 48 weeks were similar to 24 weeks, and significant at all doses compared to baseline and placebo (Table 1). Metabolite 2-hydroxybutyrate (2-HB), which was not significantly affected by retatrutide treatment at 24 weeks, was lowered significantly compared to baseline after 48 weeks of treatment (Table 1). Changes in uric acid were also consistent with week 24 (Table 1).

### Metabolic remodeling after 24 and 36 weeks of retatrutide treatment in the T2D trial population

The same targeted LC-MS/MS assays covering 139 small polar metabolites were applied to 621 fasting plasma samples from 213 participants in the T2D trial at baseline, 24, and 36 weeks. Baseline patient characteristics were similar across all treatment groups (Table S1 [17]). Global lipidomics was performed on 623 samples (97% overlap with metabolomics), and lipid mediators were assayed in the T2D trial only (due to sample availability constraints in the obesity trial). A summary of all significant changes is presented in the supplemental results section (17).

Metabolic changes similar to those observed in adults living with obesity or overweight reemerged in our analysis of the T2D cohort.

In direct comparison with the obesity cohort, FAO-related changes in the T2D population were substantially attenuated: 3-HB increases with retatrutide 8 and 12 mg reached only approximately one-third of the maximal change observed in participants without T2D, and the C2/C0 ratio increase was approximately half. Similarly attenuated changes in mid- and long-chain AC (C8, C10, C14:2, C14:1, C16:2) were observed at 24 weeks with 8 and 12 mg doses (Table S5 (17)).

The same mediation analysis in the T2D trial detected significant but attenuated joint mediation by 3-HB and C2/C0 (Fig. 4B). Neither biomarker had significant mediation individually. We estimated these biomarkers jointly mediated 12.7% of the weight loss response to retatrutide, which was ∼50% less in the T2D trial population relative to the obesity trial population. We note the endpoint of the obesity trial was 48 weeks posttreatment, while the T2D trial was 36 weeks, therefore we replicated the mediation analysis at 36 weeks posttreatment in the former to control for duration of treatment. The results were consistent with those at 48 weeks for the obesity trial (Fig. S4 [17]). The mediation analysis considers the difference in the magnitude of weight loss which in the T2D trial was −16.9% at week 36, compared to up to −21.5% and −24.2%, at 36 and 48 weeks, respectively, in the obesity trial (8, 10).

Similar to the obesity cohort, retatrutide treatment of the T2D population showed preferential decreases in TGs with shorter and more saturated fatty acids (Table S5 (17)). BCAA reductions were observed at 24 and 36 weeks, consistent with in-trial observations (10). Mean percent change from baseline reached −20.5%, −26.3%, and −29.0% for isoleucine, leucine, and valine, respectively, for retatrutide 12 mg at week 24 (Table S5 (17)), closely matching the obesity cohort. BCAA catabolic products decreased in parallel. A difference between populations was the robust decrease in threonine-derived metabolite 2-HB in the T2D cohort, which was not observed in the obesity trial; however, dulaglutide 1.5 mg also lowered 2-HB (Table S5 (17)). The IR-associated metabolites 2-AAA and uric acid were also significantly lowered compared to baseline at retatrutide doses of 8 and 12 mg, at both 24 and 36 weeks (Table S5 (17)).

Lastly, we measured a group of bioactive lipids that regulate various physiological processes, including inflammation, immunity, and cell signaling (28). We noted 2 species that were modestly altered with retatrutide treatment. Retatrutide was associated with lower inflammatory marker 2,3-dinor-11β-PGF2α at 24 weeks for the 8 mg dose group (average percent change from baseline of −22.1%, nominal *P*-value = .02) and just missed significance for the 12 mg group (average percent change from baseline of −19.3%, nominal *P*-value = .07). At 36 weeks, the lowering was sustained and significant for both doses (−27.1% and −26.4% for retatrutide 8 and 12 mg, respectively, nominal *P*-values < .05). Also, a potent peroxisome proliferator-activated receptor gamma agonist, 15-Deoxy-Δ12,14-prostaglandin J2, was significantly increased by 41.1% and 40.5% at week 36 for retatrutide 8 and 12 mg, respectively (nominal *P*-values = .01 and .026).

## Discussion

The magnitude of weight reduction observed with retatrutide in the obesity and T2D phase 2 trials is among the largest reported with pharmacological interventions (8, 10). Compared to bariatric surgery, multireceptor agonists are less characterized metabolically. We applied metabolomics and lipidomics to investigate retatrutide-associated biochemical changes, focusing on intermediary metabolites previously linked to cardiometabolic disease risk. Two key signatures emerged: (1) changes in FAO-related metabolites and (2) changes in IR biomarkers, including BCAAs and selective lowering of TGs enriched in short, saturated fatty acids.

The ketone body 3-HB showed the largest increase from baseline in both cohorts. These changes were not associated with ketoacidosis except for one case in the T2D trial (12 mg escalation group) (8-10). In both cohorts, increases in 3-HB, C2/C0 ratio, and medium- to long-chain ACs support activation of the mitochondrial FAO pathway (8-10, 29). We note that the carnitine shuttle and the buffering of acetyl-CoA with carnitine occur in various tissues, including muscle. Ketogenesis, on the other hand, occurs predominantly in the liver. Therefore, acylcarnitine levels in the blood may reflect the rate of FAO in peripheral tissues, not just the liver's ketogenic activity (29).

FAO activation by retatrutide is hypothesized to derive at least in part from direct GCG receptor agonism (1), with high activity particularly in hepatocytes. The rapid and robust changes are unlikely to be explained by weight reduction alone. Glucagon directly stimulates lipolysis via cAMP/protein kinase A-mediated phosphorylation of hormone-sensitive lipase and suppresses glycolysis through regulation of fructose-2,6-bisphosphate (27). This in turn lowers hepatic malonyl-CoA, relieving inhibition of CPT1 to promote FAO (25, 27).

The FAO cluster was robustly altered at 24 weeks in the obesity cohort, with attenuation at 48 weeks, suggesting early mobilization of stored lipids. MRI from a MASLD sub-cohort confirmed profound reductions in hepatic and visceral fat at week 24 (9). FAO activation mediated 23.2% of the weight reduction in the obesity cohort vs 12.7% in the T2D cohort; ∼50% less. This parallels the ∼20% greater end-of-study weight reduction achieved by participants without T2D at higher doses (8, 10). These between-population differences may reflect adaptation in metabolic flexibility. Metabolic inflexibility refers to a loss of normal regulation of the catabolism of metabolic fuels in response to variations in physiologic conditions. It is believed to play a role in the accumulation of ectopic fat and the development of IR, as well as hindering weight reduction and predisposing subjects to weight regain (30).

Further studies are needed to determine whether metabolic inflexibility contributes to differences in weight reduction between participants with obesity with and without T2D.

Retatrutide-associated FAO and ketogenesis induction may have cardiac implications, as heart failure is characterized by a shift from FAO to carbohydrate utilization (31, 32). In these circumstances, the heart increases its utilization of ketones as an energy source (33, 34). Studies have shown a beneficial effect of exogenous ketone delivery on cardiac remodeling and mitochondrial dysfunction in animal models (32). A phase 2 clinical trial of ketone therapy in subjects with heart failure with reduced ejection fraction demonstrated significant improvements in cardiac function and physical limitations (35). Further studies are needed to test the utility of retatrutide for enhancing cardiac health in participants with obesity with and without T2D, and with enhanced cardiovascular disease risk. The effect of retatrutide on cardiovascular outcomes is being evaluated prospectively in adults with obesity, with or without T2D, in the TRIUMPH-OUTCOMES trial (NCT06383390).

Ketosis may also have neuroprotective effects by reducing oxidative stress and enhancing mitochondrial function, as suggested by preclinical and early clinical studies in epilepsy and neurodegenerative diseases (36-39). Similarly, further studies are needed to evaluate any potential neuroprotective effects of retatrutide.

A second major metabolic signature was observed for analytes associated with IR and cardiometabolic disease. In a pattern distinct from the FAO-related metabolite cluster, change in the IR-associated metabolite cluster was robust at both 24 weeks and study endpoints, in both the obesity and T2D trial populations. The sustained improvements beyond initial metabolic adaptations may reflect targeting of pathways causally linked to IR and T2D progression. Improvements in these health outcomes have been attributed to several factors beyond changes in fat oxidation, including reduced ectopic fat deposition and reduced systemic inflammation, which can improve insulin signaling (40, 41).

Our lipidomics analysis revealed preferential lowering of TGs with shorter and more saturated acyl side chains, aligning with Framingham cohort data identifying these species as biomarkers for future T2D risk (42). This preferential lipolytic pattern may contribute to glycemic control effects. A similar pattern was observed with tirzepatide (43), suggesting that biased lipolysis may be driven by shared GIP receptor/GLP-1R agonism or by substantial weight reduction. Comparative studies are needed to deconvolute these effects.

BCAA and catabolic product changes were observed in both populations. Lowering of circulating BCAAs in animal models of obesity by feeding of BCAA-restricted diets or activation of branched-chain ketoacid dehydrogenase (BCKDH), the rate-limiting step in the BCAA catabolic pathway, has a number of beneficial effects including lowering of liver and skeletal muscle fat storage, improvement in glucose homeostasis and insulin sensitivity, and a shift to a more healthy profile of FAO in the heart (44-47).

Similar BCAA changes with tirzepatide were associated with improved insulin sensitivity and reduced T2D progression risk (43). A phase 3 tirzepatide study in obesity with prediabetes demonstrated markedly lower T2D progression risk (48).

We also observed lowering of 2-HB, 2-AAA, and uric acid. 2-HB derives from 2-ketobutyrate (2-KB), an alternative BCKDH substrate (49, 50). 2-KB is generated from methionine/threonine catabolism and glutathione anabolism in response to oxidative stress. Circulating 2-HB has been linked to impaired glucose tolerance, elevated free fatty acids, and oxidative stress (49-51). 2-HB may be more specifically linked to T2D-associated metabolic dysregulation than to obesity per se (51), which may explain why 2-HB changes in the obesity cohort emerged only at study endpoint, whereas dose-dependent lowering was already robust at 24 weeks in the T2D cohort, a population-specific difference warranting further mechanistic investigation.

2-AAA, lowered with retatrutide, was originally described as a driver of insulin secretion that may lead to β-cell exhaustion and failure (52). It has since been shown to impair insulin signaling in tissues such as liver, skeletal muscle, and adipocytes, leading to dysregulated gluconeogenesis. Lipidomic analyses revealed an interplay of 2-AAA with ACs and BCAAs in obesity and related comorbidities, suggesting 2-AAA plays a role beyond glucose metabolism (53).

Circulating uric acid was also lowered; uric acid induces fat accumulation and IR via NLRP3 inflammasome activation (54). Hyperuricemia can lead to gout; it is an independent risk factor for T2D and closely associated with IR in observational studies as well as meta-analyses (55). Circulating levels are an indirect reflection of intracellular uric acid, which is postulated to be causal for IR (56). Glucagon promotes renal uric acid excretion (57), though without urinary data we cannot confirm whether increased excretion accounts for the observed reduction in circulation.

The metabolic signature reported here with retatrutide treatment shares notable similarities with changes observed after bariatric surgery. Metabolomic studies of patients undergoing Roux-en-Y gastric bypass (RYGB) and sleeve gastrectomy (SG) have consistently reported early increases in ketone bodies, including 3-HB, reflecting activation of FAO in the immediate postoperative period (7, 58, 59). Bariatric surgery also produces robust and sustained decreases in circulating BCAAs and their catabolic products, which have been associated with improved insulin sensitivity and reduced T2D risk (7, 58, 60, 61). Reductions in circulating TGs and lowering of amino acids involved in gluconeogenesis, such as alanine, have similarly been documented following both RYGB and SG (58, 62). Of particular interest, the temporal pattern of metabolic remodeling with retatrutide—an early, robust FAO response that attenuates over time, alongside sustained improvements in IR biomarkers—mirrors the metabolic trajectory described after bariatric surgery, where ketogenesis-related changes tend to be transient while BCAA and TG improvements persist (7, 59). These parallels suggest that retatrutide may recapitulate key aspects of the metabolic reprogramming achieved by bariatric surgery through pharmacological multireceptor agonism, though direct comparative studies are needed to determine whether the underlying mechanisms are shared or distinct.

Study limitations include a relatively small sample size, which limits the generalizability to a broader population of subjects living with obesity, with or without T2D. The results of these post hoc analyses should be considered as hypothesis-generating and not definitive. Independent validation of the findings has not yet been conducted. Deconvolution of differential effects of the agent studied here, the tri-agonist retatrutide, relative to effects of mono-agonists, or GIP receptor/GLP-1R or GLP-1R/GCG receptor dual-agonist agents will require direct, side-by-side and dose-controlled comparative studies. Deconvolution of direct or indirect effects due to weight, diet, or lifestyle changes on the metabolic signature measured would also require additional investigations. Insufficient information was collected during the trials to look for specific associations. Also, the phase 2 clinical trials were not designed to measure energy intake or energy expenditure and their potential contributions to weight reduction. Additional mechanism-of-action studies in future clinical trials and preclinical studies will be needed to further characterize the translational impact of retatrutide on nutrient disposition and energy expenditure. We also note that all samples analyzed in this study were obtained from fasted individuals, potentially obscuring important observations with retatrutide in recently fed individuals as well as in kinetics of metabolism. In addition, in the results and discussion, we present both differences and similarities between obesity and the T2D trial populations, but acknowledge these are based on independent statistical analyses. While the use of 3 complementary metabolomics platforms provided relatively broad analyte coverage in this study, we acknowledge that some classes of analytes were not represented in the resultant datasets, thus possibly obscuring other relevant metabolic pathways modulated with treatment. Finally, the analytical strategy adopted provides a relative quantitation of metabolites and lipids compared to pooled calibrators and internal standards. While this approach is commonly used in large-scale targeted metabolomics and includes multiple quality controls, the changes we observed are not expressed as absolute concentrations, somewhat limiting clinical interpretability and stratification across populations.

## Conclusion

This study provides a broad overview of circulating metabolites and lipids following treatment with retatrutide in a cohort of subjects living with obesity, with and without T2D. This work affords an opportunity to tie the previously reported robust clinical outcomes with changes in systemic biochemical pathways known to underlie cardiometabolic disease pathophysiology. Our findings suggest that retatrutide treatment was accompanied by an early modulation of circulating markers of FAO, which appeared to be attenuated in participants living with T2D compared to participants living with obesity without T2D, possibly contributing to differences in efficacy of the weight reduction in the 2 cohorts. In contrast, retatrutide was associated with changes in metabolites and lipids associated with T2D risk and metabolic dysregulation similarly in both populations, regardless of T2D status, in a direction consistent with improved metabolic health, and strongly associated with reduction in cardiovascular risk independent of intervention. Further research is needed to improve our understanding of retatrutide effects on obesity and related comorbidities and the underlying mechanisms, whether involving direct effects on target tissues or improvement in body weight, glucose levels, energy expenditure, and other cardiovascular risk factors.

## Data Availability

Eli Lilly and Company provides access to all individual participant data collected during the trial, after anonymization, with the exception of pharmacokinetic or genetic data. Data are available to request 6 months after the indication studied has been approved in the United States and the EU and after primary publication acceptance, whichever is later. No expiration date of data requests is currently set once data are made available. Access is provided after a proposal has been approved by an independent review committee identified for this purpose and after receipt of a signed data sharing agreement. Data and documents, including the study protocol, statistical analysis plan, clinical study report, and blank or annotated case report forms, will be provided in a secure data sharing environment. For details on submitting a request, see the instructions provided at www.vivli.org.

## Clinical trial information

The phase 2 trials were registered at ClinicalTrials.gov with numbers NCT04881760 (obesity trial) and NCT04867785 (T2D trial).
